# Tactile-like corpuscles in gastric mucosa: a case report

**DOI:** 10.1186/1477-7819-4-39

**Published:** 2006-07-05

**Authors:** Sadia Salim, Beiqing Liu, Mandana Mahmoodi, Haider Asad, Steve J Hou

**Affiliations:** 1 Department of Pathology and Laboratory Medicine, Drexel University College of Medicine, Philadelphia, PA, USA; 2 Department of Pathology, St. Peter's Hospital, 315 South Manning Blvd, Albany, NY, USA

## Abstract

**Background:**

The presence of tactile corpuscle-like structures in Schwannomas, Neurofibromas and Neuroid Intradermal Melanocytic Nevi is well-documented. We report a case describing the presence of such structures in the lamina propria of grossly normal gastric mucosa.

**Case presentation:**

A 30 year-old male underwent esophagogastrectomy for carcinoma. Examination of hematoxylin and eosin stained sections reveal tactile corpuscle-like structures in the mucosa adjacent to the main tumor mass.

**Conclusion:**

This is a rare phenomenon and a literature search revealed only one paper describing such structures in the benign colonic mucosa of a colectomy done for carcinoma. We did not come across any cases in the literature describing such structures in gastrointestinal specimen resected for reasons other than carcinoma. To our knowledge this would be the first case reporting the existence of tactile corpuscles-like structures in gastric mucosa.

## Background

Tactile corpuscle-like structures, also called tactoid bodies, wagner-meissner-like corpuscles, pacinian corpuscles-like structures and pseudomeissnerian corpuscles are exclusively found in peripheral nerve sheath tumors such as schwannoma, neurofibroma [1-6] and neuroid intradermal melanocytic nevi [7]. In the classification by Krucke (1974), neurofibromas with these structures were considered a distinct histological subtype (type III neurofibromas) [8]. The presence of such structures in plexiform neurofibromas has been associated with von Recklinghausen's disease [6]. The cells comprising these structures are arranged in a whorled or lamellated arrangement and are considered to be of perineural and schwannian nature [3,4].

## Case presentation

A 30-year-old caucasian male presented with progressive dysphagia for the past few months. The patient had a history of smoking one pack per day and heavy alcohol use for many years. His medical history was significant for hypertension. An endoscopic biopsy revealed an adenocarcinoma at the gastroesophageal junction. Gross examination of the subsequent esophagogastrectomy specimen revealed a 5.5 × 3.0 × 1.2 cm fungating tumor, located at the gastroesophageal junction. The remaining gastric and esophageal mucosa appeared unremarkable. Sections of the tumor, random sections of uninvolved mucosa and other pertinent sections were examined after hematoxylin and eosin (H&E) staining.

### Pathological findings

**Figure 1:**
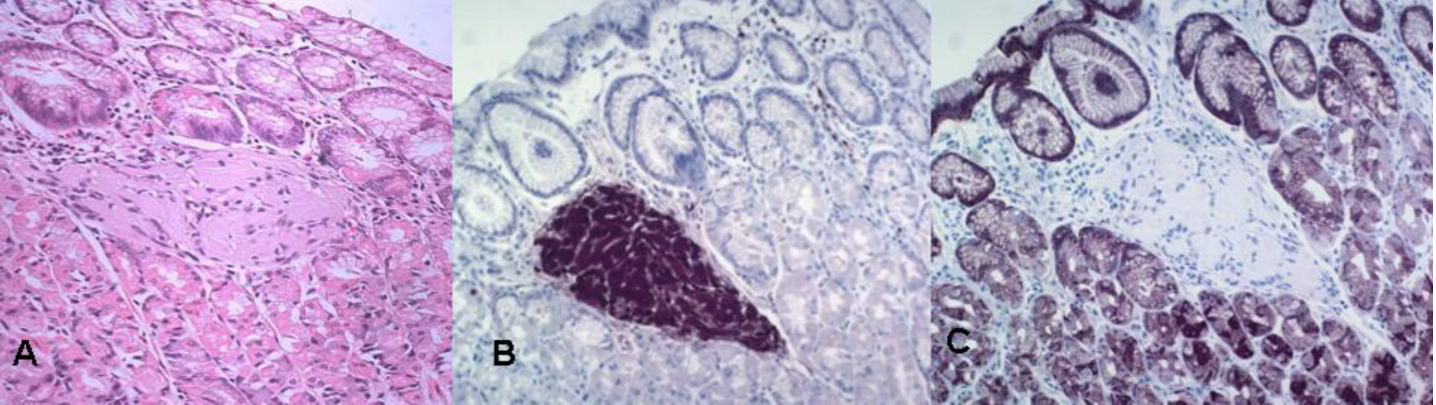
Tactile-corpuscle like structure in gastric mucosa 1**A. **The lamellar architecture of these ovoid corpuscles is seen surrounded by normal appearing gastric mucosa. (hematoxylin-eosin 20×) 1**B. **Strong S-100 immunohistochemical staining of cytoplasm (20×) 1**C. **Negative immunohistochemical staining with EMA while the surrounding gastric mucosa is staining strongly positive (20×)

On histopathology the tumor was diagnosed as poorly differentiated adenocarcinoma. Light microscopic examination of random gastric sections revealed multiple small corpuscle like structures in the lamina propria close to the top of the gastric pits (Figure 1A). These corpuscles were round to oval in shape ranging in size from 99–330 microns in diameter approximately. Histologically they were comprised of elongated slender cells with slightly eosinophilic cytoplasm and an oval to pale nucleus and were tightly packed in a lamellated arrangement with a well-circumscribed contour. Whorled and parallel-running fibers could also be identified. Adjacent gastric tissue had no significantly histopathological change.

To determine the nature of the cells forming these lamellated structures, we examined related cell membrane markers by using biotin-avitin immunohistochemical methods. The slides were stained with the following immunohistochemical stains: S-100, epithelial membrane antigen (EMA), neuron specific enolase (NSE), nerofilament protein (NF), glial fibrillary acid protein (GFAP), myelin basic protein (MBP) and CD 57 (Dako Corporation, California).

These cells stained strongly positive with mouse monoclonal antibodies to human S-100 (Figure 1B), lightly positive by the antibody to NSE and stained negative with antibodies against EMA (Figure 1C), GFAP, NF, MBP and CD 57.

**Figure 2:**
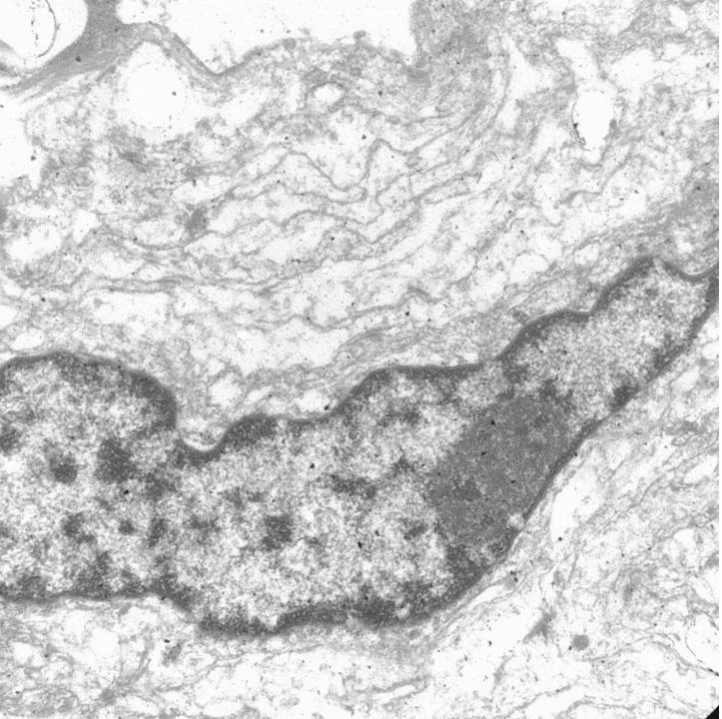
Ultrastructural study. Electron micrograph reveals the peripheral nucleus, elongated cell processes. (2780×)

**Figure 3:**
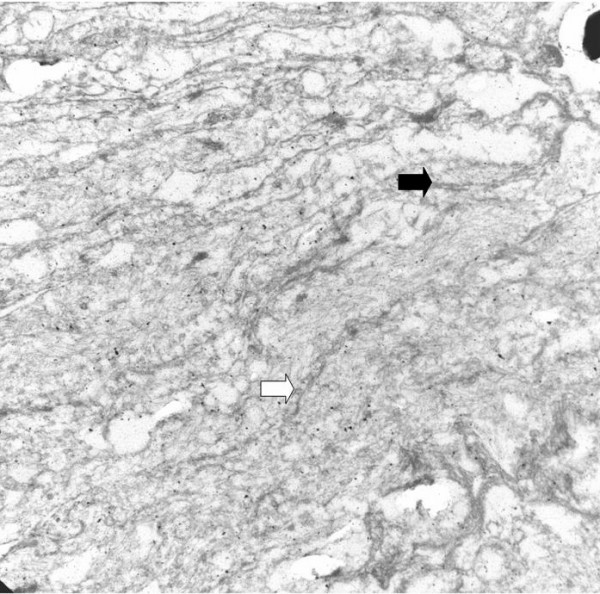
Ultrastructural study. Lamellated processes with caveolea (white arrow) and rudimentary intercellular junctions (black arrow) (2780×).

Ultrastructural studies were performed on formalin fixed tissue. Although the tissue was inadequately preserved for such an analysis, it revealed structures similar to the ones described in neurofibromas [1,5]. The structures were composed of cells with peripherally located nuclei and delicate lamellated cytoplasmic processes. Surface cavoelae, and rudimentary intercellular junctions [1,2] described in such structures were not seen very clearly due to poor preservation (Figure 2 &3).

## Discussion

Tactile corpuscle-like structures are neurogenic lamellated formations composed predominantly of Schwann cells or perineural cells or both. They are almost exclusively associated with certain types of nerve sheath tumors and intradermal nevi [9,10].

In the current report, we describe the presence of such structures in the lamina propria of grossly normal gastric mucosa in a patient with gastroesophageal adenocarcinoma. The morphologic features of these corpuscles are compatible with those described in associated with nerve sheath tumors and the single case of colonic carcinoma. Positive immunohistochemical stains for the nerve cell marker (S-100 and NSE) demonstrate their neurogenic origin. Along with morphologic observations, this immunohistochemical staining pattern supports that these corpuscle-like structures found incidentally in our case, are similar to the tactile corpuscle-like structures described in peripheral nerve sheath tumors or dermal nevi and are of Schwann-related cell origin [4]. In fact, several other authors have come to the conclusion that these structures are composed predominantly of Schwann cells [3].

Based on the clinical information, the patient in our case has no history of nerve sheath tumor or tactile corpuscle-like structure related disorder. We do not believe that there is any relationship between these structures and adenocarcinoma in term of tissue origin and since carcinoma is the most common reason for colectomy or esophagogastrectomy, these findings may be purely incidental. On the other hand, it is possible that these corpuscles might be acquired during the neoplastic changes, for which we cannot provide any evidence at present and further analysis of such cases is essential. Could these represent embryonic remnants of sensory nerve system in viscera or have the potential to be a source for the development of neurogenic tumors in lamina propria of gastric mucosa need to be answered. It is worth being mentioned here that gastric submucosal tumors of neurogenic origin with Schwann cell element are not as infrequent as we thought. Yagihashi *et al*., demonstrated with immunohistochemical techniques that some gastric submucosal tumors, originally diagnosed as leiomyomas and leiomyosarcoma, were actually the tumors of neurogenic origin [11]. The most important diagnostic relevance of these findings is to make histopathologists aware that such an entity exists and should not be misinterpreted as a ganglioneuromatous lesion or other neurogenic lesions in small biopsies.

## Competing interests

The author(s) declare that they have no competing interests.

## Authors' contributions

**BL **provided the case for this case report, **SA **reviewed literature and had the central responsibility in preparing and coordinating writing of the manuscript. **MM, HA **reviewed literature and helped in preparation of the manuscript. **JSH **offered valuable experience in writing of this report. All authors read and approved the final manuscript.
